# Psychological Predictors of Sexual Intimate Partner Violence against Black and Hispanic Women

**DOI:** 10.3390/bs8010003

**Published:** 2017-12-27

**Authors:** Brianna Preiser, Shervin Assari

**Affiliations:** 1 Department of Psychiatry, University of Michigan, Ann Arbor, MI 48109, USA; preisebj@med.umich.edu; 2 Center for Research on Ethnicity, Culture and Health, School of Public Health, University of Michigan, Ann Arbor, MI 48109, USA

**Keywords:** ethnic groups, intimate partner violence, sexual coercion, depression disorder, anxiety disorder, problem alcohol use, African Americans, Blacks, Hispanics

## Abstract

**Background:** Although various types of intimate partner violence (IPV) tend to co-occur, risk factors of each type of IPV may differ. At the same time, most of the existing literature on risk factors of IPV among minorities has used a cross-sectional design and has focused on physical rather than sexual IPV. We conducted the current study to compare Black and Hispanic women for psychological predictors of change in sexual IPV over time. **Methods:** Using data from the Fragile Families and Child Wellbeing Study (FFCWS), this study followed 561 Black and 475 Hispanic women with their male partners for four years. Independent variables included male partners’ depression, anxiety, problem alcohol use, and male-to-female physical and psychological IPV perpetration. The dependent variable was sexual IPV reported by female partners, measured at baseline, two years, and four years later. Covariates included age, income, marital status and education level. We used a multi-group latent growth curve model (LGCM) to explain intercept, linear, and quadratic slopes, which represent the baseline, and linear and curvilinear trajectories of male-to-female sexual IPV, where groups were defined based on ethnicity. **Results:** Psychological IPV was associated with sexual IPV at baseline among both ethnic groups. The male partner’s depression was a risk factor for an increase in sexual IPV over time among Black but not Hispanic women. Anxiety, problem alcohol use and physical IPV did not have an effect on the baseline or change in sexual IPV over time. Psychological IPV was not associated with an increase in sexual IPV over time in either ethnic group. **Conclusions:** There is a need for screening of sexual IPV in the presence of psychological IPV among minority women. There is also a need for screening and treatment of male partners’ depression as a strategy to reduce sexual IPV among Black women.

**Background:** Although various types of intimate partner violence (IPV) tend to co-occur, risk factors of each type of IPV may differ. At the same time, most of the existing literature on risk factors of IPV among minorities has used a cross-sectional design and has focused on physical rather than sexual IPV. We conducted the current study to compare Black and Hispanic women for psychological predictors of change in sexual IPV over time. **Methods:** Using data from the Fragile Families and Child Wellbeing Study (FFCWS), this study followed 561 Black and 475 Hispanic women with their male partners for four years. Independent variables included male partners’ depression, anxiety, problem alcohol use, and male-to-female physical and psychological IPV perpetration. The dependent variable was sexual IPV reported by female partners, measured at baseline, two years, and four years later. Covariates included age, income, marital status and education level. We used a multi-group latent growth curve model (LGCM) to explain intercept, linear, and quadratic slopes, which represent the baseline, and linear and curvilinear trajectories of male-to-female sexual IPV, where groups were defined based on ethnicity. **Results:** Psychological IPV was associated with sexual IPV at baseline among both ethnic groups. The male partner’s depression was a risk factor for an increase in sexual IPV over time among Black but not Hispanic women. Anxiety, problem alcohol use and physical IPV did not have an effect on the baseline or change in sexual IPV over time. Psychological IPV was not associated with an increase in sexual IPV over time in either ethnic group. **Conclusions:** There is a need for screening of sexual IPV in the presence of psychological IPV among minority women. There is also a need for screening and treatment of male partners’ depression as a strategy to reduce sexual IPV among Black women.

## 1. Background

Although both genders commit intimate partner violence (IPV) against their partners, male-to-female IPV is more common than female-to-male IPV [1]. There are two major reasons why we need more research on risk factors of sexual IPV (i.e., forced sex in intimate relationships) against minority women. First, most of the research on IPV has focused on psychological (IPV in the forms of stalking, psychological aggression, and verbal abuse that are not physical or sexual) and physical forms of violence [2,3,4,5,6]. As a result, our knowledge of psychological and behavioral risk factors of sexual IPV among ethnic minority women is very limited [7]. Second, the majority of the existing literature on psychosocial risk factors of IPV among minorities has used a cross-sectional design.

Sexual IPV includes a wide range of experiences, from coercion to unwanted sexual activity by a partner to more severe forms, such as rape. Up to 21% of women experience sexual IPV [8]. Women who experience forced sex have poor psychological, physical, and sexual health [8]. De Visser et al. [8,9] explained that the impact of sexual IPV goes beyond sexual function and includes psychosocial distress, substance use, anxiety, depression, and suicide. Women who have experienced sexual IPV are also at risk for mental health issues and relationship problems [10].

Based on the literature, sexual, psychological, and physical IPV tend to co-occur [11,12,13]. There are studies that suggest various forms of IPV may have similar risk factors [14]. There are also studies suggesting that the risk factors are specific to the type of IPV [15,16]. There is a need for additional research on the links between various types of IPV [17].

Exposure to sexual coercion, threats of violence, and physical violence often co-occur in women [18]. Victims of other types of violence (e.g., physical and psychological) are more likely to experience sexual IPV [19]. The explanation for this relationship is that sexual, psychological, and physical aggression tend to co-occur [10]. A study examining a community sample reported that 95% of women with a history of physical violence, compared to only 41% of women without such history, have been subjected to sexual IPV. Based on the same study, 97% of women with and 42% of women without a history of psychological violence had experienced sexual IPV [16]. Although male partners’ poor mental health increases the risk of IPV against female partners [20,21], most studies have interpreted psychopathology as a consequence rather than a risk factor for IPV [10,22,23,24,25]. Although it is only reported in a small minority of studies, there is evidence suggesting depression is a predictor of IPV perpetration, a finding that is still present after controlling for heavy drinking and illicit drug use [21]. Men and women with high depressive symptoms more frequently commit various forms of IPV [26,27]. Although depression may be more strongly associated with IPV victimization among women, it is more closely associated with IPV perpetration among men [28].

While research on the topic is limited, we have reason to believe that anxiety acts as a possible risk factor for perpetration of IPV [20,29,30]. Various aspects of negative emotionality (i.e., anxiety, anger, and hostility) are shared risk factors between IPV and general criminal behaviors [31]. Anxiety disorders also predict physical aggression perpetration [15].

Alcohol misuse also increases the risk of IPV perpetration among men [21,32,33,34]. Across types of IPV, male partners’ problem alcohol use may be the strongest risk factor for violence perpetration [35], considering even the effect of illicit drug use [20]. The effects of alcohol on IPV remains significant after controlling for effects of socio-demographics, mental health, personality disorders, and comorbid violence [20]. Up to 80% of IPV perpetrators use alcohol. The risk of violence after alcohol consumption is approximately 10 times higher for people with alcohol use disorder [36]. Meta-analyses have suggested that problem alcohol use has a stronger role on male-to-female than female-to-male IPV [33,37]. Most of this research, however, focuses on other types of IPV, outside of sexual IPV [38].

Using a dyadic approach that includes both partners as informants [39], the current study compared Black and Hispanic women for the effects of male partners’ depression, anxiety, problem alcohol use, and non-sexual IPV on the trajectory of sexual IPV reported by the female partner.

## 2. Methods

### 2.1. Design and Settings

This study used interview data from Waves 2 through 4 of the Fragile Families and Child Wellbeing Study (FFCWS), an ongoing longitudinal, population-based cohort, which began in 1998 [40,41,42]. The FFCWS followed about 5000 children born in large U.S. cities between 1998 and 2000. Almost three-quarters of the families enrolled to the FFCWS were composed of unmarried households. These mostly unmarried parents and their children are called “fragile families” as they are at a greater risk of separating and living in poverty than traditional families. The core FFCWS consists of interviews with both male and female partners at the birth of their child, and again when children were at ages one, three, five, nine, and 15. The study has collected extensive economic, behavioral, and health information and generated several hundred publications on different disciplines and outcomes. A list of publications is available for review [43]. The study data are publicly available for research use and analysis online [44].

### 2.2. Ethics

The study was approved by Institutional Review Board Committees at Princeton University and Columbia University. Verbal and written informed consent was obtained from participants at each interview, and all participants were compensated for their involvement in the study.

### 2.3. Participants and Sampling

The FFCWS used a random sampling method from families in 20 U.S. cities with populations of 200,000 or more. A detailed description of sampling strategy and interview protocol in the FFCWS is available elsewhere [45]. The FFCWS has oversampled non-married couples [45]. As non-marital unions are less stable than marital unions, a large number of male partners (at baseline) were not living with the female partner in subsequent waves. For instance, by wave 2, fewer than half of male partners were residing in the home with their female partner.

### 2.4. Analytical Sample

We limited the current analysis to couples in which the female partner was either Black/African American or Latino/Hispanic. As a result, 1036 of 4898 total participating couples from different ethnic groups who completed the baseline of the study were entered into the current analysis.

### 2.5. Process

Data used here were limited to the parental core interviews, interviews with the mother and father reporting on child health and development, and their own romantic relationships with the other parent and marriage attitudes. The term “parental core interview” is used to distinguish interviews with parents from interviews with a primary caregiver, which may include caregivers other than parents [46]. Participating men and women were interviewed at baseline, and then 1 (our baseline), 3 (our wave 2) and 5 (our wave 3) years later. As data on IPV has been not measured at baseline, the current study did not use baseline data and included data on IPV from years 1, 3 and 5.

## 3. Measures

Male-to-female sexual IPV. Sexual IPV was assessed by asking female partners “How often does the father (of your baby) force you to have sex/do sexual things?” Response items included never (0), sometimes (1), or often (2). This item was adapted from the Spouse Observation Checklist [47] and studies by Lloyd [46]. It has been frequently used to measure sexual IPV [48,49,50,51,52]. IPV questions were selected from the Conflict Tactic Scale [53], which was adapted from items previously used in partner violence research [54,55].

Male-to-female physical IPV. Physical IPV was assessed by asking female partners two questions on a 3-point scale (“never”, “sometimes”, or “often”), regarding how often fathers carried out behaviors toward the female partner (e.g., slapping, kicking, hitting) and were adapted from the Conflict Tactics Scale (CTS-2) for adults [53,56]. The original and revised Conflict Tactics Scales [53,56] have been the most commonly used research tool to measure domestic violence. The 1996 version includes separate measures for psychological, physical, and sexual IPV. The physical violence items of the CTS are still most widely used for assessing levels of domestic violence [57].

Major Depressive Disorder (MDD). The Composite International Diagnostic Interview-Short Form (CIDI-SF) was used to measure MDD [58]. The CIDI-SF is a standardized instrument designed using DSM-III-R criteria (American Psychiatric Association, 1994). CIDI-SF determines the probability that the respondent would be diagnosed with major depression, if given the full CIDI interview. MDD is indicated by feelings of depression or anhedonia experienced for most of the day, every day, for at least 2 weeks. Participants were classified as likely to have MDD if they endorsed the screening items (0 = no, 1 = yes) and 3 or more depressive symptoms (e.g., losing interest, feeling tired, change in weight).

Generalized Anxiety Disorder (GAD). The CIDI-SF was used to screen for symptoms consistent with a diagnosis of GAD [58]. The diagnosis is based on DSM-III-R criteria (American Psychiatric Association, 1994). The CIDI-SF has acceptable reliability and validity [58]. GAD is indicated by a period of six months or more when an individual feels excessively worried or anxious about more than one thing, more days than not, and has difficulty controlling their worries. Common symptoms include being keyed up or on edge, irritability, restlessness, having trouble falling asleep, tiring easily, difficulty concentrating and tense or aching muscles. Subjects were classified as having generalized anxiety disorder if they met full diagnostic criteria based on the CIDI-SF (0 = no, 1 = yes).

*Problem Alcohol Use.* Heavy/problem alcohol use was defined as five or more drinks during a single day over the past month (coded “1”). This measure approximates heavy drinking according to the definition by the National Institute on Alcohol and Alcoholism, i.e., five or more drinks in a single day for men [59].

Covariates. Control variables measured separately for male and female partner at baseline (wave 1). Covariates included age, education level, and minority status. We also measured family income and relationship status at the family level (reported by women).

Main outcome. We used the following single item to measure sexual IPV: “How often does he (father of your baby) force you to have sex/do sexual things?” Sexual IPV was measured at baseline, and two and four years after baseline. This conceptualization is consistent with the definition of sexual IPV provided by de Visser, et al. [8] who do not limit sexual IPV to rape and penetration, but include incidents of being forced into unwanted sexual activity by their partners.

## 4. Analysis Plan

We used SPSS 20 (IBM Inc. Armonk, NY, USA) for univariate and bivariate analyses. For bivariate associations, we calculated Pearson’s correlations between control variables, alcohol use, depression, anxiety, physical IPV, psychological IPV, and sexual IPV.

We used AMOS for multivariable analysis. We ran multi-group latent growth curve models, with groups defined based on ethnicity of the female partner. In our models, we tested paths from depression, anxiety, alcohol use, physical IPV, psychological IPV and control variables to intercept, linear slope, and quadratic slope of sexual IPV.

The chi square test, comparative fit index (CFI), root mean square error of approximation (RMSEA), and chi square to degrees of freedom ratio were considered as fit indices. A CFI of higher than 0.95, RMSEA of lower than 0.06 and chi square test to degrees of freedom ratio less than 2 were indicative of good fit [60]. While variables measured at baseline did not have missing values, variables measured at subsequent waves had up to 10% of values missing.

## 5. Results

From a total sample of 1036 minority women, 561 were Black and 475 were Hispanic. For Black females, the spouse/partner was also Black in 89% of cases, and Hispanic in 5% of cases. For Hispanic females, the spouse/partner was also Hispanic in 87% of cases and Black in 3% of cases. Education level of partner/spouse was lower among Hispanic females than Black females. The majority of both groups were either married or cohabiting at baseline. The rates of partner/spouse endorsing criteria for depression and anxiety was the same for both groups and was reported as about 4% for MDD and less than 1% for GAD. In both groups, less than 5% reported experiences of physical IPV, and about 2–3% of respondents reported some degree of sexual IPV at baseline. The proportion of female partners who reported sexual IPV generally remained stable over time (Table 1 and Table 2).

Mean age at baseline was 26 and 25 among Black and Hispanic women, respectively. Male partners were 3 years older than women, with an average age of 29 and 28 in Blacks and Hispanics, respectively (Table 3).

Our bivariate analysis showed positive associations between sexual, physical, and psychological IPV at baseline. MDD, GAD, and alcohol use were not significantly correlated with sexual IPV at baseline. Age and education level of the partner, as well as household income and relationship status were not correlated with sexual IPV (Table 4).

### 5.1. Sexual IPV against Black Women

**Figure 1:**
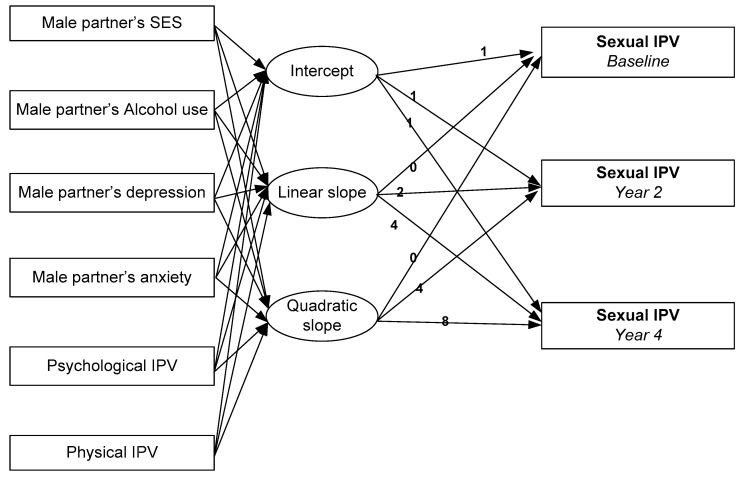
Diagram representing latent growth curve model in our study. IPV: intimate partner violence.

Model 1, which was conducted among Black women and their partners, showed an excellent fit to the data [*X*^2^ = 97.906, df = 63, *p* = 0.003, CFI = 0.972, CMIN/DF = 1.554, RMSEA = 0.031, 90% CI = 0.018–0.043]. Based on this model, MDD in male partner (at baseline) was associated with a larger linear slope for trajectory of sexual IPV during four years of follow up (B = 0.035, *p* = 0.012). Psychological IPV at time 2 was also associated with higher sexual IPV at time 2. Male partners’ age, education level, income, ethnicity, GAD, problem alcohol use and physical IPV were not significantly associated with baseline, linear, or quadratic slopes of sexual IPV over time (Figure 1).

### 5.2. Sexual IPV against Hispanic Women

Model 2 corresponds to Hispanic women and their partner /spouse. The model showed a good fit to the data [*X*^2^ = 141.681, df = 59, *p* = 0.000, CFI = 0.901, CMIN/DF = 2.401, RMSEA = 0.054, 90% CI = 0.043–0.066]. Based on this model, psychological IPV at wave 2 was associated with a higher sexual IPV at wave 2 (B = 0.174, *p* = 0.000). Male partners’ age, education level, income, ethnicity, MDD, GAD, problem alcohol use and physical IPV were not significantly associated with sexual IPV at wave 2 or linear or quadratic slopes of sexual IPV over time (Figure 1).

## 6. Discussion

This study showed similarities and differences in psychological predictors of sexual IPV against Black and Hispanic women. A similar association between psychological and sexual IPV was found for both Black and Hispanic women. Male partners’ depression was associated with a worse trajectory of sexual IPV over the follow-up period among Black, but not Hispanic women.

We found ethnic variation in the effects of depression in male partner, meaning the effect was present in Blacks but not Hispanics. Ethnic variations in the associations between psychosocial risk factors and behavioral health outcomes are shown across domains [61,62,63,64]. This finding may be simply due to cultural differences in presentation and manifestation of depression [65]. One explanation for this difference may be high comorbidity between depression and impulse control [66] particularly in minorities and poor environments [67] and low socio-economic status (SES) individuals [68,69,70]. There is literature on anger as a component of men’s depression [71]. There is also research suggesting that depression may be commonly associated with conduct or behavioral problems in some settings [72]. Blacks, individuals with low SES, and individuals with depression show higher levels of impulsivity. Finally, there is literature on high chronicity and low health care use of Blacks with psychiatric conditions, including depression [73]. We also cannot rule out the role of stigma, culture, and measurement issues in explaining the differences between Blacks and Hispanics in the effects of depression on sexual IPV.

Our finding on the effect of male partner depression on sexual IPV is supported by literature that shows psychopathology increases the risk of IPV perpetration [20,21]. Anger control problems [74] and impulsive behaviors [75] are known phenomena in patients with depression. There is, however, recent literature that suggests individuals with comorbid depression and anger control problems should be evaluated for bipolar disorders [76]. Comorbidity of depression and anger issues are more common in men than women [71]. Our knowledge of the effect of perpetrators’ poor mental health on the risk of IPV is largely derived from studies on physical or psychological IPV [77,78]. This is partly because most of the literature has conceptualized poor mental health as a consequence of IPV victimization rather than a risk factor for IPV perpetration [9,79].

Our study did not show that male partner’ problem drinking behavior had any effect on sexual IPV victimization of Black or Hispanic female partners. In a study in Russia, the odds of IPV perpetration were three times greater among men who misused alcohol than those who did not [80]. Community based studies have shown an association between problem drinking and risk of intimate partner violence in American adults [81]. Between 30 and 40 percent of men who perpetrate violence against their female partners used alcohol at the time of perpetration [82].

Furthermore, alcohol use disorder was reported as the strongest associated factor of IPV perpetration in a study conducted by Smith, Homish, Leonard, and Cornelius [83]. Compared to those whose partners never drank alcohol, women whose partners engaged in problem alcohol use were six times more likely to report IPV victimization [84]. Caetano, McGrath, Ramisetty-Mikler, and Field [85]) reported that drinking five or more drinks on occasion is positively associated with the overall occurrence of male-to-female IPV. In almost all these studies, other types of IPV, rather than sexual IPV, were the outcomes. The literature has already suggested that different forms of IPV may have different sets of protective factors and risk factors [15,16,17], and thus we argue that male partners’ problem alcohol use may not be directly related to sexual IPV among minority women.

Our study did not find that male partners’ presentation of GAD had any effect on sexual IPV against Black or Hispanic women. Again, most studies which have built our current understanding of the association between anxiety and IPV perpetration are limited to other types of IPV. In a study by Miga et al. [15], attachment anxiety predicted physical aggression perpetration. Our study does not confirm the findings of the later study.

### 6.1. Implications

Our study has four policy and clinical implications. First, as this was a community sample, rather than a clinical sample, and as minority populations have greater stigma and lower access to health care, there is a need for screening and treatment programs in the community. The first implication of our results is a need for community-based intervention to prevent of IPV in ethnically diverse communities.

Second, we found that psychological and sexual IPV co-occur, regardless of ethnicity. This finding suggests a need for combined programs which can help protect women of minority status against experiences of IPV through preventative and treatment programs. Research findings on risk factors and protective factors contributing to co-occurring physical and sexual IPC may collectively contribute to reducing ethnic disparities in the burden on IPV among women.

Third, based on our findings, there is a need to screen male perpetrators for mental health issues, and other types of IPV, particularly psychological IPV. This is an important distinction from prior approaches to identifying and addressing IPV, which have primarily focused on the screening of victims. [86] Our findings contrast with the traditional approach in which the victim, rather than the perpetrator, undergoes mental health screening [87]. This study shows that psychopathology is also a risk factor for IPV, and depression should be considered a potential cause of IPV. As the male partner’ presence may not be possible in the clinical setting, this further supports the need for community-based interventions for sexual IPV. Such community-based programs should screen both partners for depression as a cause and consequence of IPV. 

As stated, our research findings provide evidence for the importance of mental health screening of male perpetrators, in addition to female victims. A fourth implication of our study is, as psychological and sexual IPV victimization tend to co-occur among minority individuals, we propose combined programs that simultaneously address various types of IPV. Interventions aimed at the prevention of IPV may benefit from combined programs that target various forms of IPV, including sexual violence. We believe that prevention strategies may benefit from studies that focus on both of these outcomes.

### 6.2. Limitations

The current study had four significant limitations. Firstly, female victims may underreport sexual IPV, which is particularly significant for low SES and minority populations who may suffer from greater stigma in reporting. Low SES and minority status may reduce IPV disclosure [88,89,90]. Secondly, using the DSM-III is a limitation, as it does not rely on updated diagnostic criteria to address mood. Thirdly, our reliance on a single question to measure IPV is a limitation. A fourth limitation is the use of the CTS-2, which may provide different results across cultural groups and holds limited clinical utility if it is not combined with other sources of information gathering (i.e., psychometric measures or interview). In our study, CTS-2 was not administered in conjunction with clinical interviews, which could provide additional contextual data regarding violence in the family [91]. The CTS’s external validity has also been raised as a concern. Dobash and Dobash [92] have argued that, within the CTS, certain behavioral acts are open to interpretation (e.g., throwing an object at your partner) and the context behind the act (e.g., retaliation or self-defense). However, the CTS-2 is still the most widely used measure in IPV research. Another critique of the CTS is gender symmetry [92]. The CTS focuses on behavioral descriptions of conflict (e.g., punching) and avoidance of the words “abuse” and “rape” which could be interpreted differently by respondents [93]. However, the CTS has been used across cultures [94]. All the above raise concerns about the reliability and validity of the CTS measure in ethnically diverse populations. As a result, the current findings may be threatened by measurement bias. These limitations are partially a result of the data being collected by the FFCWS, which did not primarily focus on IPV in data collection and goals.

### 6.3. Future Research

Additional research is needed to understand why, how, and when women of diverse ethnic minority groups are exposed to a combination of psychological and sexual IPV. A suggested first step would be testing psychological IPV as a possible mediator for the effect of risk factors on sexual IPV. In addition, there is a need to test the efficacy of interventions meant to improve the mental health of male partners as a strategy to prevent male-to-female IPV. Future research should add clinical interviews and more contextual data along with standard measures [91].

### 6.4. Conclusions

Black and Hispanic women showed common and unique psychological determinants of sexual IPV. Psychological IPV was similarly associated with sexual IPV among both ethnic groups. Male partners’ depression was a risk factor for sexual IPV among Black but not Hispanic women. Combined programs that jointly screen for sexual and non-sexual IPV are needed among minority women, regardless of their ethnicity. There is also a need for attention to male partners’ psychopathology in the presence of sexual IPV reported by Black women.

## Figures and Tables

**Table 1:** Descriptive characteristics of male partners’ socio-demographics and mental health.

Characteristics-	Blacks (*n* = 561)		Hispanics (*n* = 475)	
	*n*	%	*n*	%
**Race and Ethnicity**				
Hispanic	27	4.81	415	87.37
Black	504	89.84	14	2.95
**Education**				
Less than high school	127	22.64	246	51.79
Completed high school or equivalent	224	39.93	115	24.21
Some college	160	28.52	85	17.89
College or graduate level	48	8.56	24	5.05
**Relationship Status at Baseline**				
Married	223	39.75	228	48.00
Romantic Cohabiting	249	44.39	223	46.95
Romantic Some Visit	41	7.31	13	2.74
Romantic No Visit	47	8.38	11	2.32
**Relationship Status at Year 2**				
Married	263	46.88	278	58.53
Romantic Cohabiting	228	40.64	185	38.95
Romantic Some Visit	33	5.88	6	1.26
Romantic No Visit	37	6.60	6	1.26
**Relationship Status at Year 4**				
Married	310	55.26	309	65.05
Romantic Cohabiting	193	34.4	151	31.79
Romantic Some Visit	23	4.10	5	1.05
Romantic No Visit	35	6.24	10	2.11
**Anxiety**				
No	499	88.95	442	93.05
Yes	3	0.53	3	0.63
**Depression**				
No	481	85.74	426	89.68
Yes	21	3.74	19	4.00
Problem Alcohol Use *				
0 day	185	67.27	117	45.00
1 day	27	9.82	54	20.76
2 day	23	8.36	39	15.00
4 day	18	6.55	29	11.15
8 day	14	5.09	13	5.00
10 or more days	8	2.91	7	2.69

* How many days in the past month the partner had 5+ alcoholic beverages; IPV: intimate partner violence.

**Table 2:** Descriptive statistics of intimate partner violence among female partners.

	Blacks (*n* = 561)		Hispanics (*n* = 475)	
	*n*	%	*n*	%
**Physical IPV Score at Baseline**				
0	553	98.57	455	95.79
1	4	0.71	12	2.53
2	3	0.53	6	1.26
**3**	1	0.18	0	0.00
**Sexual IPV**				
**Baseline**				
Often	2	0.36	0	.000
Sometimes	13	2.32	7	1.47
Never	546	97.33	468	98.53
**2 Years Later**				
Often	5	0.89	1	0.21
Sometimes	8	1.43	4	0.84
Never	548	97.68	470	98.95
**4 Years Later**				
Often	1	0.18	3	0.63
Sometimes	12	2.14	11	2.32
Never	548	97.68	461	97.05

IPV: intimate partner violence.

**Table 3:** Descriptive statistics of male partners’ socio-demographics and mental health.

	Blacks (*n* = 561)			Hispanics (*n* = 475)		
	Range	Min-Max	Mean (SD)	Range	Min-Max	Mean (SD)
Female parent age	27	15–42	25.98 (5.90)	28	15–43	25.38 (5.56)
Male parent age	37	16–53	28.75 (7.47)	37	16–53	27.63 (6.39)
Household income	133,750	0–13,3750	32,673.38 (28,541.69)	131,508	0–131,508	30,015.92 (26,757.84)
Problem alcohol use	31	0–31	1.38 (3.84)	30	0–30	1.73 (3.43)
Physical IPV at baseline	3	0–3	0.02 (0.21)	2.00	0–2	0.05 (0.27)
Psychological IPV at baseline	8	0–8	0.70 (1.17)	7.00	0–7	0.82 (1.21)
Sexual IPV baseline	2	1–3	2.97 (0.19)	1	2–3	2.99 (0.12)
Sexual IPV at 2 years	2	1–3	2.97 (0.22)	2	1–3	2.99 (0.12)
Sexual IPV at 4 years	2	1–3	2.98 (0.16)	2	1–3	2.96 (0.21)

IPV: intimate partner violence.

**Table 4:** Correlation matrix between male and female partners’ characteristics.

	1	2	3	4	5	6	7	8	9	10	11	12	13	14	15	16
Female partner age	1	0.727 **	0.326 **	0.369 **	0.329 **	−0.299 **	−0.023	0.050	−0.044	−0.086	−0.003	0.011	0.020	−0.050	−0.046	−0.075
Male partner age	0.727 **	1	0.218 **	0.214 **	0.251 **	−0.274 **	−0.030	−0.008	−0.051	−0.067	−0.052	−0.029	0.051	−0.024	−0.034	−0.039
Household income	0.102 *	0.131 **	1	0.515 **	0.402 **	−0.326 **	−0.037	0.066	−0.018	−0.030	−0.125 *	−0.045	−0.142 **	−0.023	0.059	0.029
Female partner education	0.192 **	0.116 *	0.286 **	1	0.529 **	−0.290 **	0.038	−0.010	−0.028	−0.128 **	−0.011	−0.011	−0.125 **	0.025	0.093 *	0.063
Male partner education	0.127 **	0.211 **	0.311 **	0.494 **	1	−0.315 **	0.026	−0.039	−0.050	−0.100 *	−0.069	−0.038	−0.078	−0.001	−0.008	0.015
Relationship	−0.122 **	−0.146 **	−0.107 *	−0.077	−0.134 **	1	−0.054	0.074	−0.043	−0.001	0.031	0.125 **	0.065	−0.007	0.011	−0.051
Hispanic men	−0.029	−0.034	−0.159 **	−0.220 **	−0.203 **	0.016	1	−0.669 **	0.104 *	0.046	0.099	0.015	0.042	0.036	0.033	−0.016
Black men	−0.039	−0.069	0.070	0.054	0.019	0.032	−0.458 **	1	−0.062	0.002	−0.057	0.009	−0.039	0.008	−0.049	0.020
Anxiety	−0.020	0.038	−0.020	−0.012	0.051	0.055	−0.051	−0.015	1	0.242 **	−0.022	−0.009	0.111 *	0.013	0.012	0.012
Depression	−0.062	−0.020	−0.007	−0.001	−0.007	0.087	−0.085	0.089	0.390 **	1	0.018	0.114 *	0.057	−0.017	−0.011	−0.136 **
Alcohol	−0.009	−0.037	−0.109	−0.070	−0.062	0.116	0.012	−0.032	−0.045	−0.025	1	0.068	0.192 **	0.023	−0.002	0.045
Physical IPV	−0.035	0.010	−0.088	−0.044	−0.070	−0.015	−0.046	0.059	−0.016	−0.041	0.039	1	0.288 **	−0.160 **	−0.061	−0.085 *
Psychological IPV	0.029	0.064	−0.108 *	−0.062	−0.100 *	0.059	−0.032	−0.019	0.101 *	0.064	0.172 **	0.323 **	1	−0.159 **	−0.119 **	−0.055
Sexual IPV baseline	0.052	0.033	−0.033	−0.047	−0.009	−0.022	0.059	−0.082	0.010	0.027	−0.068	−0.235 **	−0.321 **	1	0.230 **	0.423 **
Sexual IPV 2 years	−0.093 *	−0.111 *	−0.028	0.014	0.034	−0.011	−0.037	0.017	0.008	0.021	−0.409 **	−0.042	−0.324 **	0.394 **	1	0.316 **
Sexual IPV 4 years	−0.010	−0.006	−0.018	0.091 *	0.056	0.045	0.054	−0.086	0.014	0.036	−0.109	−0.220 **	−0.200 **	0.302 **	0.359 **	1

IPV: intimate partner violence. Statistics related to Black female partners are reported in the top diagonal. Statistics related to Hispanic female partners are reported in the bottom diagonal. * Correlation is significant at the 0.05 level (2-tailed). ** Correlation is significant at the 0.01 level (2-tailed).
